# Job demand and job resource factors explaining stress and job satisfaction among home care nurses – a mixed-methods sequential explanatory study

**DOI:** 10.1186/s12912-023-01568-3

**Published:** 2023-10-27

**Authors:** Anu-Marja Kaihlanen, Salla Ruotsalainen, Visa Väisänen, Laura Corneliusson, Tiina Pesonen, Timo Sinervo

**Affiliations:** Finnish Institute for Health and Welfare, P.O. Box 30, Helsinki, FI-00271 Finland

**Keywords:** Home care, Nurses, Job demand, Job resources, Stress, Job satisfaction

## Abstract

**Background:**

Increasing home care has been seen as a way to respond to the growing care needs of the aging population. To secure a sufficient number of nurses to provide home care, it is essential to identify and take into account the factors related to their well-being and job satisfaction. This study examined associations of both objective and subjective job demands and resources with stress and job satisfaction among nurses working in home care.

**Methods:**

This study used a mixed-methods sequential explanatory design. First, quantitative data was collected with a survey, followed by a qualitative survey with open-ended questions. Linear regression analyses and qualitative content analysis with an inductive approach were used to analyze the quantitative and qualitative data, respectively. Joint display in a form of a table was used to integrate the results.

**Results:**

Of the objectively measured job demands and resources, higher proportion of direct care time and higher number of interruptions were associated with higher stress in the fully adjusted models. Of the subjective measures, higher time pressure, role conflicts and disruptions were associated with higher stress. Higher time pressure, role conflicts, and disruptions in the workday were associated with lower job satisfaction, whereas higher care continuity and having more autonomy were associated with higher job satisfaction. The results of the qualitative study, in which the nurses described their experiences of their working week, partly explained and confirmed the results of the quantitative study but were also contradictory in some respects.

**Conclusions:**

Many demands, both subjectively experienced and objectively measured in terms of work organization, may undermine home care nurses’ well-being and enjoyment at work. A severe nursing shortage combined with a growing number of clients is the reality of home care, significantly increasing the risk of burnout and turnover among employees. Strategies must be developed urgently to ensure sufficient personnel in home care. For example, investments should be made in opportunities for autonomous planning of work, and promotion of good cooperation and functionality of work teams.

**Supplementary Information:**

The online version contains supplementary material available at 10.1186/s12912-023-01568-3.

## Background

Provision of home care has emerged as a vital component of health and social care delivery because population aging is a phenomenon that globally challenges the operation of health and social service systems [1]. It is estimated that in most countries, the share of the population over 80 years old will multiply within the next decades [2], significantly increasing the need for health and social care services [3]. As an effort to limit growing care costs, and to extend the opportunities for the aging people to live and manage at home, many countries have aimed to increase the provision of home care services [2, 4]. Political trends and priorities have also created pressure to increase the provision of home care, as home care is seen as a beneficial alternative to institutional care for the service users, families, and society as a whole [5, 6]. In Finland, home care is placed as a priority form of care for older people [7]. On a national level the aim is that majority of older persons are living in their own homes [8].

As the need for care for older people continues to increase, so does the need for qualified nursing staff [3]. The global shortage of nurses is one of the major problems in relation to the growing care needs of aging population, and it is predicted to worsen in the future [9]. Even if home care is a priority in Finland, and there have been some efforts aiming to increase the number of personnel and attract more personnel, the number of care staff has not increased accordingly [10]. Furthermore, commitment and sufficiency of personnel was noted as problem in a study assessing the evolution of home care services in Finland [11]. Also globally nursing staff shortages have been found to be particularly problematic in the home care sector, where difficulties in attracting and retaining qualified professionals have been reported [12, 13]. An insufficient number of care personnel increases the strain and exhaustion among the home care employees [14], which consequently reflects on their job satisfaction [15] and on the quality of care [13, 16]. Although the provision of home care has significantly increased in recent years, majority of the research on the wellbeing and strain of nurses working in older people care has still focused on nursing homes or assisted living facilities. To promote the future availability and retention of nursing workforce in home care, more up-to-date knowledge is needed on the factors that can cause stress and affect job satisfaction.

The Job Demand-Resource model (JD-R) has been used for decades in various disciplines to explain how different aspects of work are connected to employees’ wellbeing and organizational outcomes [17]. According to the JD-R model, the physical, psychological, social, or organizational aspects of the job, such as workload, time pressure, or work interruptions, are connected to the stress and burnout experienced by the employee. Whilst the resources of the job, such as received support at the workplace or task autonomy, they can reduce stress and are seen as unique predictors of work engagement that can also buffer the effects of job demands on the employee's well-being [18]. The strength of the JD-R model is its flexibility, as the model can incorporate a wide range of job characteristics and thus be tailored and applied to different work environments [19]. The JD-R model has been tested and used in various health care contexts [20–22], but to a lesser extent in studies concerning home care [23].

In previous research, there has been some effort to identify factors related to the stress and job satisfaction of nurses working in home care. According to the findings, factors such as reasonable workload, autonomy, flexible time schedules, and supportive work relationships may have positive effects on home care workers stress levels and job satisfaction, and facilitate their intentions to remain at work [24–26]. In turn, experiencing time pressure (e.g., insufficient working hours to provide optimal care for a large number of clients), constant work interruptions, role conflicts, or having less autonomy have been suggested to increase stress and job dissatisfaction among home care workers [14, 26, 27].

Although some knowledge on the wellbeing of home care nurses is available, it is still relatively scarce compared to research done in other nursing environments. An earlier study by Ruotsalainen et al. [14] have been conducted in a Finnish home care setting, however, it was based on a small dataset from two cities and a rather limited number of work units. Moreover, this earlier study and other previous studies have mainly relied on subjective self-reported data. A significant gap in knowledge lies in understanding the connection between objectively measured job demands and resources (the way work is actually planned and executed) and employee outcomes [28], and within the home care context a need for the use of different indicators to test the assumptions of the JD-R model has been established [23]. It is also notable that existing studies have mainly been carried out before the COVID-19 pandemic, which is known to have had a significant impact on nurses’ job demands and available job resources both in older people care and home care contexts [29, 30]. Hence, to fill the existing research gap, the purpose of this study was to examine the associations between various job demands and job resources on the stress and job satisfaction of nurses working in home care, using both objective and subjective measurements. We hypothesised that a) both objectively measured and subjectively experienced job demands would be associated with higher stress and lower job satisfaction, and b) both objectively measured and subjectively experienced job resources would be associated with lower stress and higher job satisfaction among nurses. In addition to this, the quantitative study was combined with a qualitative element, the aim of which was to provide complementary and more detailed information on the demands and resources experienced by home care nurses. The information obtained can help home care organisations to choose targeted actions to meet the identified job demands of home care nurses. Leaders can use the results in evidence-based decision-making and resource allocation to create a more supportive and efficient work environment, which can increase job satisfaction and retention.

The research questions were as follows:

1. Which objective job demands and objective job resources associated with stress and job satisfaction among home care nurses? (Quantitative).
2. Which subjective job demands and subjective job resources associated with stress and job satisfaction among home care nurses? (Quantitative).
3. What job demands and job resources home care nurses describe as having experienced during their work week? (Qualitative).
4. Do nurses’ descriptions about their experienced job demands and job resources help to explain the results of the quantitative phase of the study? (Qualitative and quantitative).

**Fig. 1:**
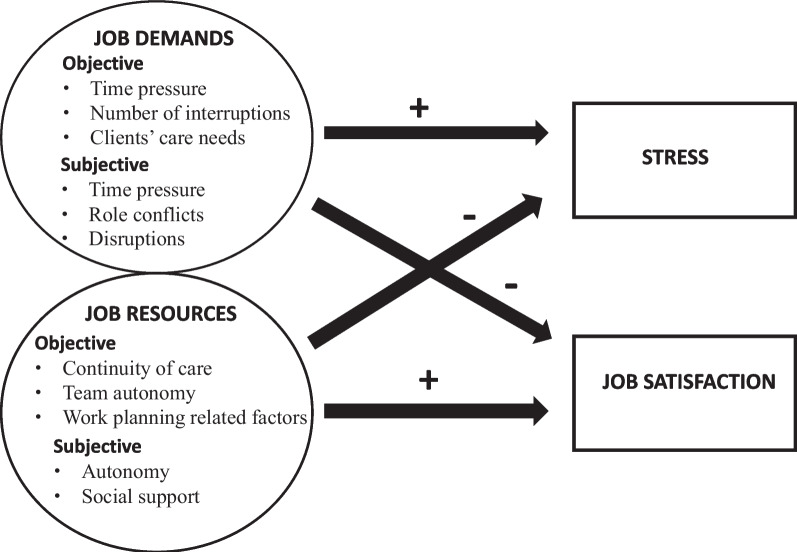
Variables used in the quantitative study and the assumed directions of associations

The associations to be tested in the quantitative study and the assumed directions (+/-) of the associations are demonstrated in Fig. 1.

## Methods

### Design and setting

A mixed-methods sequential explanatory design was chosen because it enabled a more in-depth understanding of the factors related to stress and job satisfaction of home care nurses than a qualitative or quantitative study alone [31]. A quantitative study was later followed by a qualitative study, the results of which aimed to help to explain and give more detailed information on possible observed associations. Good Reporting of A Mixed Methods Study (GRAMMS) guideline was used in reporting.

In Finland, home care is publicly funded and combines home nursing and home help services. Home care provided by the private sector is very small in scale, covering 15%. Private home care services are typically services outsourced by Wellbeing service counties and are regulated by the same legislation and service fees than the public services. In 2020, approximately 16 percent of citizens over 75 years of age received regular home care services [32]. In home care, the nursing staff's tasks are focused on assisting in daily activities and responding to the clients’ other care needs [33]. Other services, such as cleaning or meal and grocery shopping services, can be acquired via home care providers, however, they may need to be paid out-of-pocket. Several home care organizers are using Enterprise Resource Planning systems (ERP) to organize the care of the clients and employees’ workdays. ERP-systems aim to distribute the clients efficiently for the employees, considering the routes and tasks that need to be conducted at the client’s home.

This study was conducted in publicly funded home care organizations (*N* = 17) located in five different regions across Finland, including rural and urban areas.

### Participants and data collection

The quantitative data was gathered with paper and online survey in October 2021. The anonymous paper survey was sent to 17 home care units with 431 workers, including nurses and other professionals. The total number of surveys sent was based on the estimation given by the supervisors of the work units, since we did not have the precise number of individuals working in the units. Only those respondents who had a nursing degree (practical nurse or registered nurse) were included in this study (*N* = 386), due to dissimilarities in job descriptions between other professionals (such as therapists). The survey included two parts: [1] Time Measurement Survey, where the employees filled in their client visits and tasks conducted during one week, and [2] Wellbeing Survey, which included questions regarding wellbeing and job satisfaction. The online survey (Manager Survey) was sent to the unit managers (*N* = 17) after the paper survey. In the survey, unit managers were asked to provide information, for example, regarding the ERP systems and team autonomy in their organization. In addition to the survey data, we utilized the Finnish Institute for Health and Welfare registers to obtain information about client characteristics (based on clients’ assessments of Resident Assessment Instrument, RAI).

The qualitative data was gathered from the same home care units (*N* = 17) with an anonymous online questionnaire three months after the quantitative surveys (February 2022). The questionnaire consisted of open-ended questions in which the employees were asked to freely describe their experiences of their workweek, for example, how they had experienced the functionality of the work community, work arrangements, leadership, workload, job satisfaction or the effects of the ERP system on work. A link to the questionnaire was distributed to the employees via their supervisors.

All the questionnaires used in the study are presented in Supplementary file 1.

### Instruments

#### Stress

Stress was measured with a single-item question, previously validated in a study from Elo et al. [34]: “*Stress means feeling tense, restless, nervous, or anxious, or being unable to sleep at night because one’s mind is troubled all the time. Do you feel stressed today?*” [34]. Participants answered on a five-point Likert scale (ranging from 1 = ‘not at all’ to 5 = ‘very much’).

#### Job satisfaction

Job satisfaction was measured with a single-item question “*In general, I am very satisfied with my job*”, originally adapted from the Job Diagnostic Survey, [35] and answered with a five-point Likert scale (ranging from 1 = ‘fully disagree’ to 5 = ‘fully agree’). Previous studies have shown good validity of single-item measure for job satisfaction [36] and a strong correlation between multiple-item scales and single-item job satisfaction [37].

#### Objective job demands

Objective job demands included (1) time pressure (including proportion of direct care time and breaks), (2) number of interruptions, and (3) clients’ care needs. The proportion of direct care time, breaks and interruptions were calculated from the Time Measurement survey, where staff members had filled all their work tasks, the time they had spent conducting the tasks, total amount of time allocated for breaks and whether interruptions had occurred during their workday [33]. Clients’ care needs were assessed using the Case Mix Index, retrieved from the clients’ RAI-assessments. The values of the Case Mix Index are based on the Finnish RUG-III/18-classification system (Resource Utilization Groups) [33]. The Case Mix Index divides clients into clinical diagnostic groups, which have their own CMI values [38]. Higher CMI values indicate clients requiring more resources compared to the average client, with the baseline value being 1 [38]. CMI 0.80 means that the client needs 20% less resources than average client and 1,20 indicates 20% more resources.

#### Subjective job demands

The subjective job demands included (1) time pressure, (3) role conflicts, and (3) whether something disrupted the workday.

Time pressure [39] was formed of two items (Cronbach’s Alpha 0.81) asking how much the issues in question had disturbed, worried or burdened the respondent: “*I do not have enough time for clients*”; “*I do not have enough time to perform work properly*”. Items were rated on a five-point scale (ranging from 1 = ‘not at all’ to 5 = ‘very much’).

Role conflicts [40] were formed of the items “*In some situations, I had to act against the rules and principles in order to get my job done” and “I get tasks without having enough resources or tools to carry them out”.* These were rated on a five-point scale (ranging from 1 = ‘never’ to 5 = ‘very often’). Cronbach’s Alpha for these items was 0.68.

The possible disruptions during the workday were assessed by asking the participants whether their workday had “went as planned”, “went nearly as planned” or “something disrupted the course of the workday”. Responses were dichotomized as ‘workday went as planned’ = 0 and ‘something disrupted the workday’ = 1, which included the responses of “went nearly as planned” and “something disrupted the course of the workday”.

#### Objective job resources

The objective job resources included (1) continuity of care (i.e., possibility to work with familiar clients), (2) team autonomy, and (3) other work planning related factors.

Continuity of care was calculated based on the number of different clients the employee had visited during their workweek, divided by the number of workdays during the week. Each client was counted once, regardless of the number of weekly visits. A lower value indicated higher continuity of care (the employee provided more care to familiar clients).

Team autonomy was formed by seven items (Cronbach’s Alpha 0.76) that described whether the work team was able to decide autonomously about their work planning, client visits, recruitment, use of substitute workers, working methods, care of clients, and participation into trainings. The items were rated on a scale ranging from 1 ‘not at all’ to 4 ‘team can decide fully autonomously’.

The other work planning related factors included questions about whether “*an Enterprise Resource Planning system (ERP) takes the teams into account when planning the workday*” and whether “*the teams can influence the ERP’s plan (e.g., making changes into client visits or modifying the client list if an unexpected situation occurs)*”. Answer options were 1 ‘yes’ or 2 ‘no’.

#### Subjective job resources

Subjective job resources included (1) autonomy at work and (2) social support. The item regarding autonomy at work [41] was “*At my work, I can make a lot of independent decisions”* and the item related to social support [41, 42] was “*I receive support and help from colleagues when needed*”. Items were rated on a five-point scale (ranging from 1 = ‘fully disagree’ to 5 = ‘fully agree’).

### Data analysis

We applied multivariate linear regression when analyzing quantitative data. First, the associations of each independent variable were tested separately with the outcome variables (stress and job satisfaction) adjusted for age, sex, and occupational status (practical or registered nurse). Second, the variables with a significant association to the outcome variables were included in the multivariate model. The multivariate models were tested separately for objective and subjective variables. Finally, all independent variables (both objective and subjective) that were significantly associated with the outcomes in the previous models, were tested together in the final model. No multicollinearity was found in the models (Variance Inflation Factors, VIF, values were less than 2, for each independent variable). The analyses were conducted using SPSS, version 28, and the statistical significance was defined at *p* < 0.05.

Qualitative content analysis with an inductive approach [43] was employed to analyze the qualitative data. The aim of this analysis was to identify job demands and job resources experienced by nurses during their workweek. First, all reflections on their work week by the nurses (*n* = 47) were carefully read to gain an overall picture of their experiences. The length of the nurses' written responses varied from a couple of sentences to several sentences. The data was coded by extracting all the individual expressions describing any job demand or resource from each response, which were then renamed to a short form that matched the original expression as closely as possible. These codes were then grouped as subcategories (*n* = 11) based on the similarity of their content. Finally, the subcategories were divided into two main categories, distinguishing between job demands and resources. One researcher (A-MK) carried out the initial coding and categorisation independently, but the content and names of final subcategories, and their inclusion in either job demands or resources, were confirmed through collaboration with the authors, who had also familiarised themselves with the original data. The joint examination of the results obtained from the quantitative and qualitative data and the formation of conclusions were also conducted collaboratively with the authors.

## Results

First, this chapter presents the quantitative results based on linear regression analysis. Then, the results of the qualitative part are described according to the demands and resources experienced at work, and last, the mixed method results are presented as a joint display in Table 2.

### Quantitative results

#### Sample

The characteristics of the sample are presented in Table 1. The total number of participants was 386, leading to an approximated response rate of 90 percent. The majority (93%) of the respondents were female. Their mean age was 43 years, ranging between 18 and 70 years. Licensed practical nurses were the largest occupational group, consisting 88 percent of the sample. The mean score for nurses’ stress was 2.6 on a scale from 1 to 5, indicating an average level of stress. Job satisfaction of the participants was fairly high, with a mean of 3.8 on a scale from 1 to 5.

**Table 1:** Characteristics of the sample

N total	386
Age mean	43 (range 18–70)
Sex	N (%)
Female	327 (85)
Male	26 (7)
Missing/not announced	33 (8)
Occupational status	N (%)
Registered nurses	48 (12)
Practical nurses	338 (88)
Work experience (median)	5
Stress mean (SD)	2.6 (1.13)
Job satisfaction mean (SD)	3.8 (0.97)

### Associations of objective job demands and resources with stress

Of the objective job demands, variables indicating time pressure (higher proportion of direct care time, lower proportion of breaks) and higher number of interruptions were associated with higher stress in the univariate models (Table 2). When testing the objective job resources, lower care continuity (working with many different clients) was associated with higher stress in the univariate model. In the multivariate model with both objective job demands and resources, higher proportion of direct care time and higher number of interruptions were statistically significantly associated with higher stress.

**Table 2:** Linear regression analyses showing the univariate and multivariate associations of subjective/objective demands and resources with stress

Independent variables		Adjusted^a^ univariate model β(95%CI)	Adjusted^a^ multivariate model with objective variables β(95%CI)	Adjusted^a^ multivariate model with subjective variables β(95%CI)	Final model^a^ β(95%CI)
Objective demands					
Direct care time (% of workday)		0.019 (0.007‒0.03)***	0.015 (0.003‒0.026)*		0.013 (0.003‒0.022)*
Breaks (% of workday)		-0.046 (-0.08‒-0.013)**	-0.03 (-0.064‒0.004)ns		
Interruptions		0.105 (0.012‒0.198)*	0.097 (0.006‒0.188)*		0.01 (-0.074‒0.094)ns
Case Mix Index		-0.432 (-0.969‒0.104)ns			
Objective resources					
Care continuity (Working with familiar clients)		0.074 (0.006‒0.142)*	0.058 (-0.01‒0.125)ns		
Team autonomy		-0.084 (-0.366‒0.197)ns			
Possibility to influence ERP's plan		-0.017 (-0.294‒0.259)ns			
ERP takes teams into account		0.21 (-0.072‒0.492)ns			
Subjective demands					
Time pressure		0.492 (0.389‒0.595)***		0.247 (0.112‒0.383)***	0.249 (0.117‒0.381)***
Role conflicts		0.597 (0.462‒0.732)***		0.289 (0.123‒0.455)***	0.313 (0.151‒0.475)***
Disruptions		0.869 (0.61‒1.128)***		0.439 (0.169‒0.709)**	0.392 (0.128‒0.655)**
Subjective resources					
Autonomy		-0.134 (-0.284‒0.016)ns			
Social support		-0.276 (-0.4‒-0.152)***		-0.068 (-0.185‒0.049)ns	
		R Square	0.093	0.332	0.341

*p*-values: *p <  = 0.05. ** *p* < 0.01. ***p < 0.001

*ns* not significant

^a^Adjusted for age, sex and occupational status (practical vs registered nurse)

### Associations of subjective job demands and resources with stress

Of the subjective job demands, having more time pressure, role conflicts and disruptions during the workday were associated with higher stress in the univariable model. Of the subjective job resources, social support was associated with lower stress in the univariate models. In the multivariate model with both subjective job demands and resources, having more time pressure, role conflicts and disruptions, remained significantly associated with higher stress. Social support lost its significance.

Both objective and subjective variables indicating time pressure, as well as subjective role conflicts and disruptions remained significant in the final model, where all the significant independent variables from the multivariate models were tested together.

### Associations of objective job demands and resources with job satisfaction

When examining the univariate associations between job satisfaction and objective job demands, lower proportion of direct care time and higher proportion of breaks were associated with higher job satisfaction (Table 3). All variables related to objective job resources (higher care continuity, higher team independence, and more team considerate work planning) were associated with higher job satisfaction in the univariate models. In the multivariate model with both objective job demands and resources, only higher care continuity was associated with higher job satisfaction.

**Table 3:** Linear regression analyses showing the univariate and multivariate associations of subjective/objective demands and resources with job satisfaction

Independent variables		Adjusted^a^ univariate model β(95%CI)	Adjusted^a^ multivariate model with objective variables β(95%CI)	Adjusted^a^ multivariate model with subjective variables β(95%CI)	Final model β(95%CI)
Objective demands					
Direct care time (% of workday)		-0.014 (-0.023‒-0.004)**	-0.01 (-0.021‒0.001)ns		
Breaks (% of workday)		0.033 (0.003‒0.063)*	0.018 (-0.014‒0.05)ns		
Interruptions		-0.032 (-0.114‒0.051)ns			
Case Mix Index		-0.09 (-0.567‒0.387)ns			
Objective resources					
Care continuity (Working with familiar clients)		-0.11 (-0.169‒-0.051)***	-0.075 (-0.14‒-0.009)*		-0.038 (-0.089‒0.012)ns
Team autonomy		0.445 (0.204‒0.687)***	0.252 (-0.052‒0.556)ns		
Possibility to influence ERP's plan		-0.263 (-0.508‒-0.017)*	-0.066 (-0.321‒0.189)ns		
ERP takes teams into account		-0.318 (-0.568‒-0.068)*	-0.099 (-0.386‒0.188)ns		
Subjective demands					
Time pressure		-0.326 (-0.423‒-0.23)***		-0.19 (-0.304‒-0.076)**	-0.175 (-0.286‒-0.063)**
Role conflicts		-0.444 (-0.571‒-0.317)***		-0.249 (-0.39‒-0.107)***	-0.24 (-0.379‒-0.102)***
Disruptions		-0.596 (-0.83‒-0.363)***		-0.248 (-0.475‒-0.021)*	-0.237 (-0.458‒-0.016)*
Subjective resources					
Autonomy		0.474 (0.354‒0.594)***		0.427 (0.314‒0.541)***	0.435 (0.324‒0.546)***
Social support		0.169 (0.057‒0.282)**		-0.019 (-0.118‒0.08)ns	
		R Square	0.096	0.362	0.374

*p*-values: *p <  = 0.05. ** *p* < 0.01. ***p < 0.001

*ns* not significant

^a^Adjusted for age. sex and occupational status (practical vs registered nurse)

### Associations of subjective job demands and resources with job satisfaction

In the univariate models, having more time pressure, role conflicts, and disruptions in the workday (subjective demands) were associated with lower job satisfaction, whereas having more autonomy and social support (subjective resources) were associated with higher job satisfaction. In the multivariate model, all variables, except social support, remained significantly associated with job satisfaction.

In the final model that included all the significant independent variables from the previous models, higher time pressure, role conflicts and disruptions were associated with lower job satisfaction, whereas higher autonomy was associated with higher job satisfaction.

### Qualitative results

A total of 47 nurses answered the qualitative questionnaire, where they were asked to freely describe their experience of their work during the last week (such as the functioning of the work community, work arrangements, management, workload, job satisfaction etc.). The majority of the respondents were practical nurses (64%) and the rest were registered nurses (19%) or had some other job title, such as supervisor (17%). 38 percent of them were over 50 years old, 32 percent were between 40–49 years, and 22 percent between 16–39 years old. The results of the qualitative content analysis of nurses’ reflections on their workweek are presented in the following paragraphs, categorised as experienced job demands and job resources.

### Experienced job demands

Based on the analysis of the qualitative data, the main job demands experienced by the home care nurses were related to *increased time pressure*, *constant staff shortages and lack of substitutes*, and conflict related to the *unrealistically planned workload*.

The nurses often described *the increased time pressure* in connection with poor organization of work, and the excessive rush was seen as preventing the implementation of high-quality care. Nurses described that the pace of work has tightened over the years due to the increased number of clients, and they felt that too short times were systematically planned and reserved for client visits. One nurse mentioned that "we haven't had time to take coffee breaks in the afternoon for many years" and nurses also described that there was not enough time set aside for required paperwork, such as updating clients’ care plans. Lack of time for keeping or listening to situation reports between nurses at the change of work shifts were also raised up.

“There are often moments at work that feel busy, and it feels like there is not enough time to do all the work, client visits are far too short, which makes the feeling of urgency stronger, even if you take this information to the nurse in charge, you don't always have time to change the times of the visits.”

“We do not have a report at the end of the morning and evening shift, as is done in e.g., ward care. Unless you don't have time while changing your work clothes to give a situation update to a colleague on their way home from work.”

On the other hand, several nurses also described the opposite side, i.e., experiences of the absence of time pressure, and sufficient time and a feeling of lack of urgency seemed to be connected to nurse’s experience of a good work week.

“The week went well and was not busy. I didn't feel stressed at all”.

“This week was relaxed. There was enough time for clients and transitions between clients. There was no rush.”

The burden and negative effects of *constant staff shortages and lack of substitutes* came up in the responses of many nurses. They pointed out that for a long time the workload had been increased by the lack of nurses and the long absences of existing employees in home care, and the attempt to recruit substitutes had not yielded results. Due to sickness absences and the lack of substitutes, the work was left to be handled by an understaffed group of nurses. This was an especially prominent problem during the holiday seasons. Understaffing was a considerable physical and mental burden for nurses who had to constantly stretch and flex in relation to work shifts and do extra work on top of their normal workload.

“There is mental pressure due to employee shortages. Recruitment problems are worrying and have increased the mental load. The feeling has been such that you can no longer do anything.”

“Recently, due to the lack of employees, the work week is noticeably burdened by the constant need for extra shifts, i.e., double shifts, it is difficult to refuse them, even if you cannot really manage to walk 15 hours straight up and down the streets and stairs. Recovery from double shifts is slow and days off are mostly spent in bed.”

Several nurses also described stress and conflict related to an *unrealistically planned workload.* They felt that too many client visits were often planned in relation to the time given and the times reserved for client visits were not always realistic or corresponded to the client's care needs. There was hardly any room for flexibility in the planned work lists, so that any unexpected changes in the client's situation/care needs or other unplanned additional work (for example, due to the absence of another employee) lengthened the working day and increased the strain. Moreover, the schedules of client visits were not always logically planned in terms of transitions from one place to another, and the time required for transitions was not sufficiently considered.

“The times overlap and there is not enough time allocated for transitions, so the work that comes behind burdens the entire working day. The confusion of the routes also takes time from nurses and clients.”

“Sometimes the work is organized very poorly, a lot of overlapping visits. In the morning, almost all clients should be visited right away, because the need for help is so great. There are not necessarily too many visits in terms of time, although this is sometimes the case, but the biggest problem is that almost everything should be done first thing in the morning. This is very stressful and takes the joy out of work.”

### Experienced job resources

The main job resources described by nurses included *well-cooperating work community*, and *positive atmosphere in the work community*. Job resources were reduced by a *negative work atmosphere,* the *turnover of employees*, and *external work planning*.

Several nurses described their *well-cooperative work community* as a significant asset that increased job satisfaction and helped them cope with heavy work. The nurses appreciated that in the work community help was received and given when needed and that there was still flexibility among colleagues, even though it had been demanded more and more recently. Working with the same team members for a long period of time was seen as a strength.

“Our team is divided into nursing teams, my own nursing team works well, we have been together and in the same team for a long time.”

“I enjoy my work, the work community supports each other very well, help is received and given. I feel that we are going in the same direction together.”

In addition to good cooperation, nurses expressed the general *positive atmosphere in the work community* as a resource that had a significant impact on their well-being. The nurses described the positive atmosphere as being gentle, caring, and encouraging, and the open communication and confidentiality in the work community were also highlighted as factors that increased well-being and ability to manage at work.

“Pleasant and enlightened colleagues have a positive effect on being comfortable at work. The team has a confidential, open and encouraging, caring atmosphere. In the group space, you can often hear laughter and joy at joint successes.”

“It's almost always a pleasure to come to work because the work community is really nice.”

In contrast, the nurses also described how the *negative work atmosphere* was reflected in their week, indicating that work team did not always act as a resource. The high work strain was seen to have an unfavorable effect on the employees’ behavior and mood, and also the "basic negativity" of some employees was described as weakening the atmosphere of the entire work community and making teamwork more difficult.

“Fatigue in home care, where I work, is clearly visible. People are tired of work, rush, labor shortage, etc. You hear that all the time, even if things aren't that bad for you. The workload also eats away the positive atmosphere.”

Employee turnover was another factor that increased nurses’ strain and reduced available job resources for nurses. Instead of receiving support from the work community, changing employees and short-term substitutes often required resources because they had to be guided and advised, which could be difficult in a tight schedule and fast work pace. New employees and substitutes were not necessarily able to carry out all work tasks equally, which increased the workload of permanent personnel.

“The constant need for substitutes also burdens permanent employees. Substitutes must constantly be advised, and extra work has to be done, because substitutes don't e.g. make clients’ store or pharmacy orders. Many things go undone when substitutes are only “guest stars” and they often do not know all the things.”

Finally, some nurses described the strain related to *external work planning.* Control and planning of one's own work had decreased, and for some it had affected the comfort in the workplace. The nurses described the poor organization of the work and that the external work planner was not always up-to-date with the situations in the field, which appeared as illogical work plans.

“At the beginning of each shift, I myself had to rethink/rationalize the client visit list given by the operations management, so that I wouldn't have to shuttle back and forth senselessly in bad winter weather.”

“Working hours have changed so that an autonomous work list cannot be made, which makes work exhausting.”

### Mixed method results

The results of the qualitative study partially confirmed the results of the quantitative study, but in some respects, they were also contradictory. Table 4. presents a synthesis of the qualitative and quantitative data sources and serves as a summary of the findings.

**Table 4:** Joint display of quantitative and qualitative results

Job demands explaining home care nurses’ stress and job satisfaction	Summary of quantitative findings	Summary of qualitative findings
Time pressure (objective and subjective)	Both, objective and subjective, time pressure variables were associated with higher stress (standardized β coefficient 0.169, *p* < 0.05, for direct care time, and 0.249, *p* < 0.001 for subjective time pressure) Higher subjective time pressure was associated with lower job satisfaction (standardized β coefficient -0.222, *p* < 0.01)	*Confirmatory findings*: Excessive time pressure was a key load factor, which was mainly due to the large number of clients and too short planned visit times *Contradictory findings*: The lack of breaks and the underestimation of the time needed for transitions and office work in work planning (or ‘indirect care time’) were raised as a significant load factor
Role conflicts (subjective)	Role conflicts were associated with higher stress (standardized β coefficient 0.223, *p* < 0.001) and lower job satisfaction (standardized β coefficient -0.220, *p* < 0.001)	*Confirmatory*: Nurses got tasks without having enough resources (time) to carry them out. The implementation of unrealistically planned work/client lists created conflicts because planned time did not always correspond to the clients' care needs, or their need for care was at the same time (overlap of visits) *Contradictory*: The nurses did not describe having to act against the rules or operating principles to get tasks done
Interruptions (objective) and disruptions (subjective)	Interruptions and disruptions were associated with higher stress (standardized β coefficient 0.122, *p* < 0.05 and 0.187, *p* < 0.01, respectively)	*Confirmatory*: Any changes in the client's care needs or other unexpected additional work (e.g. due to the absence of another employee) disrupted and lengthened the work day and significantly increased the strain *Contradictory*: Interruptions (such as phone calls, picking up keys, waiting for an ambulance, problems with car/traffic etc.) were not raised as a stress factor
**Job resources explaining home care nurses’ stress and job satisfaction**		
Autonomy (subjective)	Autonomy at work was associated with higher job satisfaction (standardized β coefficient 0.372, *p* < 0.001)	*Confirmatory*: The current working time arrangements and external work planning had led to decreased autonomy (management and planning of one's own work), which had a negative effect on work enjoyment and well-being *Contradictory*: -
Care continuity (objective)	Poorer care continuity (working with many unfamiliar clients) was associated with higher stress in the univariate model and with lower job satisfaction (standardized β coefficient -0.150, *p* < 0.05) in multivariate model	*Confirmatory*: The realization of continuity of care (working with familiar clients) was prevented by the constant absences of employees and the lack of substitutes. The client visits of missing employees were added to the lists of those at work, which meant that new/unfamiliar clients had to be cared for continuously *Contradictory*: -
Other work planning related factors (objective)	ERP system that considered the teams and where teams were able to influence the planning of client visits was associated with higher job satisfaction in the univariate models. However, in the multivariate models, the variables lost their significance	*Confirmatory / Contradictory*: Dissatisfaction was expressed in relation to the external ERP system when it did not consider the changing situations of the teams and there was no flexibility in the work lists. Dissatisfaction was also expressed in relation to overlaps in client visits, illogicality in the planned routes, and the amount of work, which did not always correspond to the time allocated to it
Social support (subjective)	Having more social support was associated with lower stress and higher job satisfaction in the univariate models. In the multivariate models, it lost its significance	*Confirmatory / Contradictory*: A well-functioning work community, where support was received and given, as well as a positive atmosphere among employees were described as the most important asset in reducing stress and promoting well-being at work. Constant rush and heavy workload were reflected in the work atmosphere and created negativity between nurses

## Discussion

This study examined the associations between various job demands and resources on the stress and job satisfaction of nurses working in home care, using both objective and subjective measurements. In addition, we applied qualitative methods to obtain more detailed information about the demands and resources experienced by these nurses in order to explain and complement the quantitatively observed results.

The associations observed, and their directions, corresponded in many respects to the assumptions made (Fig. 1) based on the JD-R model [18]. In the fully adjusted models, we found that of the objectively measured job demands, high proportion of direct care time and having many interruptions during the workday were associated with higher stress. Of the subjectively measured job demands, experiencing more time pressure, role conflicts and disruptions during the workday, were associated with higher stress. Experiencing more (subjective) time pressure, role conflicts, and disruptions were also associated with lower job satisfaction, whereas better care continuity and perceiving more autonomy were associated with higher job satisfaction among nurses. The results of the qualitative study, in which nurses described their experiences of their workweek, largely confirmed, and helped to explain the associations observed in the quantitative study, but also offered opposing views.

According to both quantitative and qualitative datasets, experiencing time pressure, which indicates that nurses feel they have too little time for clients and to do their job properly, was a central job demand connected to nurses' stress and job satisfaction. One of the main reasons mentioned in nurses’ written responses was the ongoing and worsening labor shortage, along with the unavailability of substitute employees. This meant that the work was often done understaffed, and the sick absences and vacation seasons of others further increased nurses’ workload and time pressure in the workplace. The results are in line with the findings of an earlier study on the association between lower job satisfaction and insufficient staffing [15]. Moreover, managers in the older people care in Finland described similar problems as factors that contributed to poorer care quality in a study from Corneliusson et al. [44].

Staff shortages and time pressure are not, however, prevalent in all work units. In the future, it would be important to find out which factors are related to the personnel shortage. Is it a question of resources, work organization, or leadership? Still, the connection between the time pressure, stress and job satisfaction corresponds, quite as expected, to the results of previous studies [14, 26], reflecting the continuous nature of the situation in home care. However, since the experience of time pressure is subjective and people may experience it differently [45], this study provides important new evidence that not only the perceived rush, but also the actual and objectively measured large proportion of direct care work during the day, with little or no breaks, is a significant risk for stress and decreased job satisfaction among home care nurses. This is not only worrying in terms of workforce retention, but also client safety, because it has been suggested that it is not the time pressure per se, but rather the combination of time pressure and high stress that may increase the occurrence of adverse events [46]. In addition, working under time pressure might lead to poorer quality of care in a form of providing only basic care, instead of holistic care [47]. Previous studies among staff working in home care services have similarly highlighted the relation to clients as a factor influencing job satisfaction [48] and lack of control, role conflicts and role overload as factors influencing stress [27].

In this study, we found perceived role conflicts as another significant job demand associated with nurses’ stress and job satisfaction. Nurses shed light on this associations by describing how they were given tasks without sufficient resources (time) and that sometimes unrealistically planned work/client lists did not match clients' care needs or the timing of care needs overlapped. The nurses' writings also revealed that one of the main potential causes for these conflicts was the ERP system used in work planning, the purpose of which is to optimize the execution of the work by considering the number of employees and the clients' care needs and route planning. Dissatisfaction with the ERP system was often mentioned in connection with the experience of poor external work planning, which may help to explain why nurses who felt less opportunities to make independent decisions about their work had statistically significantly less job satisfaction compared to nurses with more autonomy. This was also recognized objectively measured, because if the team had an ERP system that took teams into account and enabled changing work plans, the nurses had better job satisfaction. When home care organizations use ERP systems to optimize the work of the home care workers, they should take into account the care workers possibilities to influence the planning of the workday. Furthermore, the ERP system should operate closer to the teams, making it possible for the users of ERP systems to be familiar with the clients’ needs and team members. Our results strengthen the view that team autonomy, and especially perceived autonomy is a particularly important factor promoting job satisfaction among home care nurses [14]. On the other hand, it was a surprise that autonomy was not related to stress in the final model [41]. Home care nurses typically work alone, and in everyday work with clients, autonomy is at a very high level. Team autonomy, however, is more related to planning of the client visits. If the teams are able to plan the visits and to increase care continuity, the stress levels might be lower. In addition, this may increase their retention in home care [49, 50].

According to our qualitative findings, a supportive and positive work community seemed to be the most central resource that helped nurses to cope with the heavy workload and other negative issues related to work. Quite surprisingly, however, social support was not significantly associated with nurses’ stress or job satisfaction except in the univariate models. The fact that the associations disappeared in the multivariate model, may indicate that although social support is perceived as crucially important, it cannot fully compensate for the potential negative effects of high job demands. On the other hand, in home care, most of the work is done alone, which may also be an explanation for why the effect of social support was not significant in the quantitative analyses. Previous research on this topic has shown somewhat conflicting results. Social relations at work have been suggested to reflect in job satisfaction of nurses working in home care [24, 26] and have a significant protective effect against stress [51] as well as turnover [49]. On the other hand, in another study social support was not related to nurses' job satisfaction [14] and interventions aimed at strengthening social support have been found ineffective in reducing home care nurses’ stress [52]. Based on our findings, efforts to promote job satisfaction and reduce stress should not solely aim to improve the team spirit of the work community, but also focus on finding ways to increase workflow and ease stressful job demands.

Our quantitative results also suggest that interruptions and sudden changes in the planned working day (disruptions) can be a significant factor increasing nurses’ stress in home care. In the nurses’ written responses, however, interruptions were not much mentioned, but mainly the sudden changes, such as extra client visit due to the absence of another employee, were perceived difficult to manage within the given working hours, because there was no flexibility in the work lists for such situations. The negative effects of interruptions may increase if the work planning is centralized and the changes in ERP systems are difficult to make. A noteworthy quantitative finding was that the more nurses were required to provide care for new/unfamiliar clients (i.e., poorer continuity of care), the lower their job satisfaction was. It has been found that taking care of several clients can increase home care nurses’ dissatisfaction at work [26] and continuity of care has been linked to better care outcomes, for example by reducing the number of events leading to client’s emergency room visits or hospital stays [53]. Moreover, a study from Tourangeau et al. [54] showed that better care continuity was associated with intentions to remain employed among home care nurses. As the aging population grows, the number of home care clients is also constantly increasing, thus more research is needed on the realization of continuity of care from the perspective of both nurses and clients, and how it could best be promoted.

### Strengths and limitations

The strength of this study is the research design that includes the use of both quantitative and qualitative methods, which expands the breadth and depth of the study, leading to more comprehensive results. We also utilised methodological triangulation by incorporating a combination of objective and subjective measures to assess theoretical assumptions. This approach enhances the study's rigor and reliability, as it allows for a comprehensive evaluation from different perspectives, increasing the confidence in the study's findings. Due to a cross-sectional nature of this study, we cannot determine any causal relationships. In addition, the sample size in the quantitative part of this study was fairly small, although the participants were from several different regions and therefore we could state that they represented the Finnish home care employees fairly well. It is however possible that the units that participated were slightly better-off in terms of their recruitment situation and the effects of the covid-19 pandemic. Perhaps the units with poorer employee situation were not able and willing to participate.

It should be noted that the qualitative data that consisted of nurses’ reflections about their work week were often quite short, and more in-depth answers could probably have been obtained by interviewing, for example. However, the number of respondents was nevertheless quite large for a qualitative study (*n* = 47), and we believe that the nurses highlighted the so-called most important and main experiences of their work week, thus, their answers formed a good picture of home care work and served the aim of the study. The trustworthiness of the study was improved by reflecting on the results in discussions with the research team that was familiar with the data, and by presenting several direct quotes from the nurses' answers in connection with the results. In terms of practical implications, the obtained results provide valuable information for home care organizations and leaders about areas that require attention. Based on the identified stressors and satisfaction factors, targeted interventions and strategies can be developed to reduce stress and enhance job satisfaction in home care. This can lead to improved well-being for employees and contribute to higher job retention rates, reducing the turnover and associated costs for home care organizations.

## Conclusions

This study suggests that nurses’ heavy workload, time pressure, role conflicts and the unpredictable nature of the work, where unexpected changes in the planned day may disrupt the provision of care, are considerable stressors in home care setting. Possibility of autonomous work planning and providing care to familiar clients, in which case the continuity of care can be realized, seemed to be the key work resources that might buffer the high work demands and positively affect nurses’ job satisfaction. The significant shortage of nursing staff can be seen in the everyday work of home care, potentially forcing nurses to compromise on the quality of care to cope with the workload. To ensure high-quality care, and to improve the chances for successful recruitment and retention of nurses in home care, urgent solutions and actions are needed to promote their working conditions and job satisfaction.

### Supplementary Information

**Additional file 1.**

## Data Availability

The datasets generated and analyzed during this study are not publicly available but are available from the corresponding author on reasonable request.

## Abbreviations

CMI: Case Mix Index
COVID-19: Coronavirus disease 2019
ERP: Enterprise Resource Planning systems
JD-R: Job Demand-Resource model
RAI: Resident Assessment Instrument
RUG: Resource Utilization Groups
VIF: Variance Inflation Factors
