# Supplementary material for: Job demand and job resource factors explaining stress and job satisfaction among home care nurses – a mixed-methods sequential explanatory study
Source: BMC Nurs. 2023 Oct 27;22:404. doi: 10.1186/s12912-023-01568-3 (PMC10612316; doi:10.1186/s12912-023-01568-3)
Supplement: Supplementary file 1 — Additional file 1. [file 12912_2023_1568_MOESM1_ESM.docx]

Supplementary file 1: questionnaires used in the study.

**Questions in the quantitative survey.**

| Outcome variables | Scale | Reference |
| --- | --- | --- |
| Stress means feeling tense, restless, nervous, or anxious, or being unable to sleep at night because one’s mind is troubled all the time. Do you feel stressed today? | 1 = ‘not at all’ to 5 = ‘very much’ | Elo et al. (30) |
| In general, I am very satisfied with my job. | 1= ‘fully disagree’ to 5 = ‘fully agree’ | Hackman et al. (32) |
| Subjective demands |  |  |
| How much the following have disturbed, worried or burdened you: |  |  |
| Time pressure (“I do not have enough time for clients”; “I do not have enough time to perform work properly”.) | 1 = ‘not at all’ to 5 = ‘very much’ | Harris (36) |
| Role conflicts (“In some situations, I had to act against the rules and principles in order to get my job done” and “I get tasks without having enough resources or tools to carry them out”.) | 1 = ‘not at all’ to 5 = ‘very much’ | Rizzo et al. (37) |
| Please assess how your workday went today. | ‘Workday went as planned’, “Workday went nearly as planned” and “Something disrupted the course of the workday”. (The last two were coded as ‘something disrupted the workday’) |  |
| Subjective resources |  |  |
| Autonomy (“At my work, I can make a lot of independent decisions”) | 1 = ‘fully disagree’ to 5 = ‘fully agree’ | Karasek (38)  Karasek & Theorell (39) |
| Social support (“I receive support and help from colleagues when needed”.) | 1 = ‘fully disagree’ to 5 = ‘fully agree’ | Karasek (38)  Karasek & Theorell (39) |

**Questions from the managers’ survey**

|  | Scale |
| --- | --- |
| Is the team able to decide autonomously of the following. |  |
| 1. Work planning 2. client visits 3. recruitment 4. use of substitute workers 5. working methods 6. care of clients 7. participation into trainings | 1 = ‘not at all’ to 4 = ‘team can decide fully autonomously’ |
| “An Enterprise Resource Planning system (ERP) takes the teams into account when planning the workday” | ‘Yes’ or ‘No’ |
| “the teams can influence the ERP’s plan (e.g., making changes into client visits or modifying the client list if an unexpected situation occurs)”. | ‘Yes’ or ‘No’ |

**Questions in the qualitative survey.**

1. Describe your experience of your work over the past week.

You can describe, for example, how you experienced the functioning of your work community, work arrangements, management, workload, and job satisfaction. You can also describe the effects of a patient record system or ERP system to your work.

1. What kind of thoughts arise when you think about your work conducted with clients during the past week? You can describe, for example, the functionality of client work, the quality of life of clients and the fulfillment of their needs.
2. What are the things that help you keep up and stay in your job?
3. If you had the power to decide, how would you change and develop your work or the care of clients?

**The Time Measurement form**

How to fill the form:
 - Fill the workday start and end times. Write the client’s full name clearly.
 - Write the start and end times of your visit with a client, and what you did with the client. It’s important to distinguish if the work was done with the patient or not (other). Remember to fill the related actions. Example: If you are with a client, write time class 1 and if you are doing medication management, write action 7. In addition, if you are helping the client with eating, add action 4.

**Time class 1**: Direct care time (client present)

| **Actions** | | | | |
| --- | --- | --- | --- | --- |
| **1** Daily hygiene, toilet | **2** Shower, bath, sauna | **3** Dressing up and helping with clothing | **4** Aiding with eating/heating food | **5** Nursing prodecures |
| **6** Medicine/drug care and follow up | **7** Cleaning up (with the client) | **8** Nursing documentation (client present) | **9** Guiding or helping the client with their matters (incl. IT) | **10** Helping/guiding relatives |
| **11** Arranging care e.g. after discharge | **12** Service needs assessment, care planning | **13** Furthering functional ability and rehabilitation, outdoors | **14** Multidisciplinary work (with the client), escorting client | **15** Guiding or briefing worker/student (client present) |

**Time class 2**: Indirect care time (client not present)

| **Actions** | | | |
| --- | --- | --- | --- |
| **1** Organizing client’s services and benefits | **2** Nursing documentation | **3** Assessment of needs for services, care planning | **4** Arranging medicine / drugs |
| **5** Contact with relative | **6** Consultation | **7** Multidisciplinary meetings |  |

**Time class 3**: Other

| **Actions** | | | | |
| --- | --- | --- | --- | --- |
| **1** Travel / transition | **2** Lunch break / other break | **3** Meetings | **4** Office work incl. ordering accessories | **5** Guiding or briefing worker/student, drug-exam and evaluation |
| **6** Research and development -related work | **7** Managerial and administrative work incl. announcements | **8** Collecting tools & clothes required (incl. maintenance) | **9** Other (cleaning office, refilling gas, car maintenance) | **10** Interruption or a work-related pause (for example: phone call) |

**Time class 4**: Remote visit

| **Actions** | | | |
| --- | --- | --- | --- |
| **1** Service needs assessment, care planning | **2** Guiding with medicine/drugs, and followup | **3** Guiding eating / and making sure clients eat | **4** Other guiding (toilet, clothing) |
| **5** Rehabilitation | **6** Social remote visit | **7** Group activity |  |
